# Afferent arteriolar responses to β,γ-methylene ATP and 20-HETE are not blocked by ENaC inhibition

**DOI:** 10.1002/phy2.82

**Published:** 2013-09-10

**Authors:** Tasuku Nagasawa, John D Imig

**Affiliations:** Department of Pharmacology and Toxicology and Cardiovascular Center, Medical College of WisconsinMilwaukee, Wisconsin

**Keywords:** Autoregulation, eicosanoids, kidney, myogenic response, purinergic receptors

## Abstract

Afferent arteriolar myogenic and tubuloglomerular feedback responses are critical for the proper maintenance of renal hemodynamics and water and electrolyte homeostasis. Adenosine triphosphate (ATP) P2X receptor activation and 20-hydroxyeicosatetraenoic acids (20-HETE) have been implicated in afferent arteriolar autoregulatory responses. Besides these two participants, members of the degenerin/epithelial Na^+^ channel (DEG/ENaC) family have been demonstrated to play a pivotal role in the afferent arteriolar myogenic response. The aim of this study was to determine if ENaC contributes to P2X receptor- or 20-HETE-mediated afferent arteriolar vasoconstriction. As previously demonstrated, afferent arteriolar diameter responses to increasing perfusion pressure from 100 to 160 mmHg were abolished by ENaC inhibitors amiloride or benzamil. Afferent arteriolar diameter decreased by 29% under control conditions and by 1% and 5% in the presence of amiloride or benzamil, respectively. The P2X receptor agonist β,γ-methylene ATP decreased afferent arteriolar diameter by 3 ± 1%, 7 ± 1%, 12 ± 2%, and 17 ± 3% in response to 0.1, 1, 10, and 100 μmol/L, respectively. ENaC inhibition did not alter the afferent arteriolar vasoconstrictor response to the P2X receptor agonist β,γ-methylene ATP. Like P2X receptor activation, 20-HETE dose-dependently decreased afferent arteriolar diameter and this vasoconstrictor response was not altered by the presence of ENaC inhibitors amiloride or benzamil. These results suggest that DEG/ENaC channels are required for afferent arteriolar autoregulatory responses; however, DEG/ENaC channels do not contribute to P2X receptor- or 20-HETE-mediated afferent arteriolar vasoconstriction.

## Introduction

Renal blood flow and glomerular filtration rate are tightly regulated at a constant level in the face of changing perfusion pressures to properly maintain whole body water and electrolyte balance (Loutzenhiser et al. 2006; Inscho 2009). This autoregulation of renal blood flow and glomerular filtration rate is impaired in disease states such as hypertension and diabetes (Griffin and Bidani 2004). Impaired renal autoregulation contributes to renal injury and progression to end stage renal disease. Autoregulation of renal blood flow and glomerular filtration rate are accomplished through the combination of the myogenic response and tubuloglomerular feedback mechanism (Navar et al. 1996; Just and Arendshorst 2003; Schnermann and Levine 2003). The myogenic response is an intrinsic property of the preglomerular afferent arteriole and results in afferent arteriolar vasoconstriction to increasing perfusion pressure or dilation to decreasing perfusion pressure (Navar et al. 1996). This simple phenomenon was observed many decades ago, but underlying vascular smooth muscle cell-signaling mechanisms are not fully understood.

A number of locally released factors, cell-signaling molecules, and cell membrane ion channels have been implicated and demonstrated to contribute to afferent arteriolar autoregulatory behavior (Navar et al. 1996; Drummond et al. 2004; Loutzenhiser et al. 2006; Inscho 2009). Locally released factors implicated in afferent arteriolar autoregulatory behavior include adenosine triphosphate (ATP) and 20-hydroxyeicosatetraenoic acid (20-HETE). ATP is locally released in response to increasing renal perfusion pressure (Navar et al. 1996; Inscho 2001; Nishiyama et al. 2001; Guan et al. 2007; Inscho 2009). ATP then decreases afferent arteriolar diameter via activation of purinogenic receptor, P2X1 (Inscho 2001; Inscho et al. 2003). A metabolite of the arachidonic acid cytochrome P450 (CYP) hydroxylase pathway, 20-HETE is a vasoconstrictor and contributes to afferent arteriolar autoregulatory behavior (Imig et al. 1994, 1996; Kaley 2000; Imig 2013). Previous studies from our laboratory demonstrated that 20-HETE participates in P2X receptor-elicited afferent arteriolar vasoconstriction (Zhao et al. 2001, 2004). Besides these locally released factors, members of the degenerin/epithelial Na^+^ channel (DEG/ENaC) family have been demonstrated to play a pivotal role in the afferent arteriolar myogenic response (Drummond et al. 2004; Jernigan and Drummond 2005; Wang et al. 2008; Guan et al. 2009). DEG/ENaC protein family members form mechanosensitive ion channels that could respond to increases in renal perfusion pressure (Awayda et al. 1995; Kellenberger and Schild 2002; Drummond et al. 2008). Interestingly, the ATP P2X receptor family has structural relatedness to DEG/ENaC protein family members and has been demonstrated to be associated with mechanotransduction processes (Di Virgilio et al. 1998; Shinoda et al. 2009; Browne et al. 2010; Kessler et al. 2011). ATP and P2X receptor activation also stimulates basolateral ENaC activity in renal epithelial cells (Zhang et al. 2007; Wildman et al. 2009). A major gap in our knowledge is whether or not ENaC participates in the afferent arteriolar vasoconstrictor response to P2X receptor activation or 20-HETE. To test this, we conducted experiments using the in vitro juxtamedullary nephron preparation and assessed the afferent arteriolar diameter response to the P2X receptor agonist β,γ-methylene ATP and 20-HETE in the presence of ENaC inhibitors.

## Material and Methods

### Animal

Experiments were approved and carried out according to guidelines of the Institutional Animal Care and Use Committee, Medical College of Wisconsin. Male Sprague-Dawley rats (Charles River, Wilmington, MA) weighing between 300 and 400 g were used. Animals were kept in a temperature-controlled environment with a 12-h light/dark cycle and were allowed free access to food and water at all times. An acclimatization period of 6 days was allowed for the rats before experimentation.

### Vascular preparation

Experiments were conducted using the in vitro juxtamedullary nephron technique, as described previously (Imig et al. 1996). Briefly, rats were anesthetized with pentobarbital (65 mg/kg body weight i.p.). The right kidney was isolated, perfused, and placed in an organ bath. The kidney was perfused with Tyrode's solution containing 6% bovine serum albumin (BSA; Calbiochem, La Jolla, CA) and a mixture of L-amino acids. Glomeruli and microvessels were exposed by careful removal of connective tissue covering the inner cortical surface. After dissection, renal artery perfusion pressure was set to 100 mmHg and continuously monitored with a pressure transducer. The organ chamber was warmed, and the tissue surface was continuously superfused with Tyrode's solution containing 1% BSA at 37°C. Afferent arteriolar diameters were measured using videomicroscopy and recorded images were analyzed using an image-shearing device. Afferent arteriolar diameters were recorded at 20-sec intervals. Steady-state diameter determinations were calculated from the average of all diameter measurements obtained during the final 2 min of each 5-min treatment period.

### Series 1: amiloride and benzamil on afferent arteriolar autoregulatory behavior

The protocol began with a 5-min control period at a perfusion pressure of 100 mmHg after an initial 10–30 min equilibration period with a Tyrode's solution containing 1% BSA. ENaC inhibitors, amiloride and benzamil were dissolved in dimethylsulfoxide (DMSO) and added to the superfusate. The superfusate was continued (vehicle DMSO control) or switched to a Tyrode's 1% BSA solution containing amiloride (10 μmol/L) or its analogue benzamil (10 μmol/L), for a 30-min incubation period (Jernigan and Drummond 2005; Wang et al. 2008; Guan et al. 2009). Afferent arteriolar diameters were assessed in response to increasing perfusion pressure from 100 to 160 mmHg (5 min each). Perfusion pressure was returned to 100 mmHg at the end of each protocol.

### Series 2: amiloride and benzamil on afferent arteriolar response to β,γ-methylene ATP

This experimental series was conducted to determine if the afferent arteriolar responses to β,γ-methylene ATP are attenuated by ENaC inhibition. Afferent arterioles were initially exposed to a vehicle DMSO 1% BSA Tyrode's control solution. Experiments involved a control period, followed by a 5-min exposure to 0.1–100 μmol/L β,γ-methylene ATP. Each vessel then underwent a 5-min recovery period in control solution before being exposed to amiloride (10 μmol/L) or benzamil (10 μmol/L) for 30 min. Next, β,γ-methylene ATP-containing solutions were introduced after ENaC inhibition and afferent arteriolar responses were reassessed.

### Series 3: amiloride and benzamil on afferent arteriolar response to 20-HETE

This experimental protocol was conducted to determine if the afferent arteriolar diameter response to 20-HETE was attenuated by ENaC inhibition. After an initial equilibration period at 100 mmHg in the control superfusate, 20-HETE was added to the superfusate and afferent arteriolar diameter responses measured. A cumulative dose-response curve to 20-HETE (0.001–1 μmol/L) was generated in containing vehicle, amiloride (10 μmol/L), or its analogue benzamil (10 μmol/L). Afferent arterioles were superfused with amiloride or benzamil for a 30-min incubation period prior to determining the response to 20-HETE. Diameter changes were monitored for 5 min at each concentration.

### Series 4: DDMS on afferent arteriolar response to β,γ-methylene ATP

The last protocol was conducted to verify that P2X activation contributes to afferent arterioles vasoconstriction via the CYP hydroxylase 20-HETE pathway. The CYP hydroxylase inhibitor N-methylsulfonyl-12,12-dibromododec-11-enamide (DDMS) was dissolved in DMSO and added to the superfusate (25 μmol/L) (Zhao et al. 2001). Afferent arterioles were initially exposed to a vehicle DMSO 1% BSA Tyrode's control solution. Experiments involved a control period, followed by a 5-min exposure to 0.1–100 μmol/L β,γ-methylene ATP. Each vessel then underwent a 5-min recovery period in control buffer before being exposed to DDMS 25–35 min. Next, β,γ-methylene ATP containing solutions were introduced after CYP hydroxylase inhibition and afferent arteriolar responses reassessed. Previous reports utilizing a similar protocol have demonstrated that repeated superfusion with β,γ-methylene ATP at least 10 min apart does not alter the response to the second application (Zhao et al. 2001).

### Statistics

Data are presented as a mean ± SEM. Difference between groups were analyzed by one-way analysis of variance (ANOVA) for repeated measures followed by Newman-Keuls multiple-range test. With the use of two-tailed test, *P* < 0.05 was considered to be significant.

## Results

### Afferent arteriolar diameter

Afferent arteriolar diameter averaged 19.2 ± 1.7 μm (*n* = 40) under control conditions at a renal perfusion pressure of 100 mmHg. ENaC inhibitors did not significantly alter afferent arteriolar diameter and diameter averaged 20.4 ± 1.3 μm (*n* = 14) in the amiloride and 19.1 ± 1.3 μm (*n* = 14) in the benzamil-treated groups. Likewise, the CYP hydroxylase inhibitor DDMS did not significantly alter afferent arteriolar diameter (18.1 ± 1.2 μm, *n* = 5). These data are in line with previously published data. The ENaC inhibition or CYP hydroxylase inhibition does not alter baseline afferent arteriolar diameter (Zhao et al. 2001; Guan et al. 2009).

### ENaC inhibition on afferent arteriolar autoregulatory behavior

**Figure 1:**
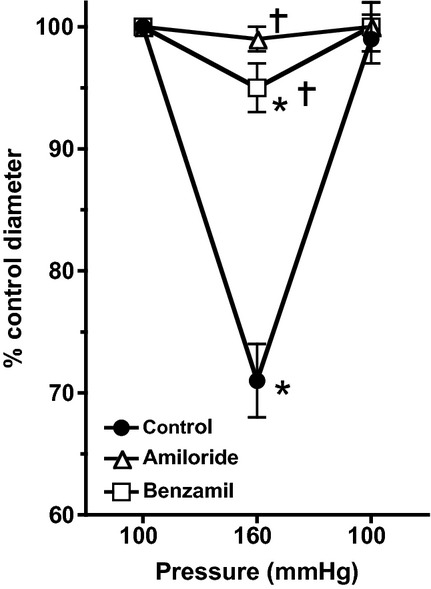
Effect of amiloride or benzamil on afferent arteriolar response to renal perfusion pressure. Afferent arteriolar responses are expressed as percent of the control diameter at 100 mmHg. Amiloride (10 μmol/L, *n* = 4) and benzamil (10 μmol/L, *n* = 4) were delivered in the superfusion solution. Values are mean ± SEM. *P < 0.05 versus control diameter in same group, †*P* < 0.05 versus control group at same perfusion pressure.

As previously published (Wang et al. 2008; Guan et al. 2009), the findings of this study demonstrate that amiloride or benzamil inhibit the afferent arteriolar vasoconstrictor response to increasing perfusion pressure (Fig. 1). Afferent arteriolar diameter decreased by 29 ± 3% when perfusion pressure was increased from 100 to 160 mmHg. In contrast, afferent arteriolar diameter decreased by 1 ± 1% in the presence of amiloride and 5 ± 2% in the presence of benzamil when perfusion pressure was increased from 100 to 160 mmHg (Fig. 1).

The findings in Figure 1 demonstrate that ENaC contributes to the afferent arteriolar response to perfusion pressure. Previous studies also support the notion ATP P2X receptor activation and 20-HETE contributes to afferent arteriolar vasoconstriction in response to increased perfusion pressure (Imig et al. 1994; Imig 2013). Unknown is the contribution of ENaC to ATP P2X receptor activation and 20-HETE-mediated afferent arteriolar vasoconstriction. Therefore, the next experimental protocols examined ENaC inhibition on the afferent arteriolar vasoconstrictor responses to P2X receptor activation and 20-HETE.

### ENaC inhibition on afferent arteriolar responses to P2X receptor activation and 20-HETE

**Figure 2:**
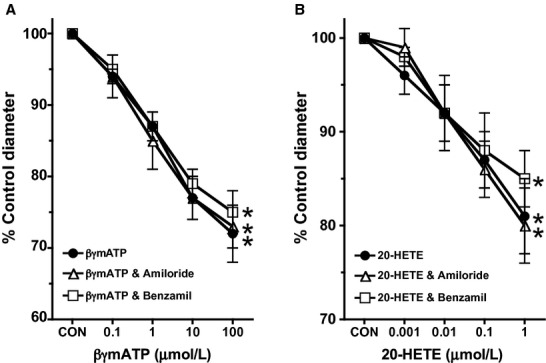
Effect of amiloride or benzamil on afferent arteriolar responses to β,γ-methylene ATP (A, *n* = 8 control, *n* = 4 amiloride, *n* = 4 benzamil) or 20-HETE (B, *n* = 7 control, *n* = 6 amiloride, *n* = 6 benzamil). Afferent arteriolar responses are expressed as percent of the control diameter at 100 mmHg. Amiloride (10 μmol/L) and benzamil (10 μmol/L) were delivered in the superfusion solution. Values are mean ± SEM.

Afferent arteriolar responses to P2X receptor agonist, β,γ-methylene ATP in the presence of the ENaC inhibition with amiloride or benzamil were evaluated. Afferent arteriolar diameter declined by 6 ± 3%, 13 ± 2%, 23 ± 3%, and 28 ± 2% in response to β,γ-methylene ATP at concentrations of 0.1, 1, 10, and 100 μmol/L, respectively. Amiloride or benzamil did not alter the afferent arteriolar vasoconstriction induced by β,γ-methylene ATP (Fig. 2A). These results suggest that ENaC inhibition did not affect the P2X receptor-mediated vasoconstriction of afferent arterioles.

Next, we determined if the afferent arteriolar constrictor response to 20-HETE depended on ENaC activation. Afferent arteriolar diameter decreased by 4 ± 2%, 8 ± 2%, 13 ± 2%, and 19 ± 3% in response to 20-HETE at concentrations of 0.001, 0.01, 0.1, and 1 μmol/L, respectively. 20-HETE afferent arteriolar vasoconstriction was not altered by the presence of the ENaC inhibitors, amiloride or benzamil (Fig. 2B). These data provide evidence that 20-HETE afferent arteriolar vasoconstriction is independent of ENaC.

### CYP hydroxylase inhibition on the afferent arteriolar response to P2X receptor activation

**Figure 3:**
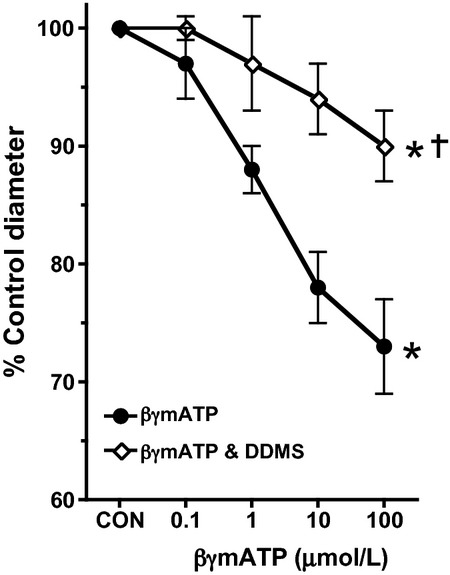
Effect of DDMS on afferent arteriolar responses to β,γ-methylene ATP. Afferent arteriolar responses are expressed as percent of the control diameter at 100 mmHg. DDMS (25 μmol/L, *n* = 5) was delivered in the superfusion solution. Values are mean ± SEM. *P < 0.05 versus control response to β,γ-methylene ATP.

Previous studies have demonstrated that CYP hydroxylase and 20-HETE inhibition attenuates the afferent arteriolar vasoconstriction to P2X receptor activation (Zhao et al. 2001, 2004). The selective CYP hydroxylase inhibitor, DDMS, was used to confirm the results of these previous studies. Afferent arteriolar diameter dose-dependently decreased in response to for β,γ-methylene ATP. DDMS greatly attenuated the afferent arteriolar response to β,γ-methylene ATP (Fig. 3). The afferent arteriolar diameter decreased by 27 ± 4% and 10 ± 3% to 100 μmol/L β,γ-methylene ATP in the absence or presence of DDMS, respectively. These results verify previous reports that CYP hydroxylase inhibition attenuates the afferent arteriolar vasoconstrictor response to P2X receptor activation.

## Discussion

Several locally released factors as well as cell membrane channels have been demonstrated to contribute to afferent arteriolar autoregulatory behavior (Navar et al. 1996; Loutzenhiser et al. 2006; Inscho 2009). These include ATP, 20-HETE, and DEG/ENaC channels (Zou et al. 1994; Inscho et al. 2003; Jernigan and Drummond 2005; Wang et al. 2008; Guan et al. 2009; Imig 2013). We previously demonstrated that 20-HETE contributed to ATP P2X receptor-mediated afferent arteriolar vasoconstriction (Zhao et al. 2001). This study determined if ENaC participates in the afferent arteriolar vasoconstrictor response to P2X receptor activation or 20-HETE. The findings of our experimental studies demonstrate that ENaC inhibition did not alter the afferent arteriolar vasoconstrictor response to P2X receptor activation or 20-HETE. We were able to confirm previous reports that ENaC inhibition attenuates afferent arteriolar vasoconstriction to increasing perfusion pressure and that CYP hydroxylase inhibition attenuates the vasoconstrictor response to β,γ-methylene ATP.

Afferent autoregulatory behavior is accomplished through the combined influences of the myogenic and tubuloglomerular feedback mechanisms (Navar et al. 1996; Loutzenhiser et al. 2006; Inscho 2009). Previous reports have demonstrated that 20-HETE contributes to the myogenic and tubuloglomerular feedback mechanisms whereas ATP P2X receptor activation contributes primarily to the tubuloglomerular feedback mechanism (Zou et al. 1994; Inscho et al. 2003; Inscho 2009; Imig 2013). Afferent arteriolar responses to P2X receptor activation require 20-HETE (Zhao et al. 2001, 2004). Mechanosensitive DEG/ENaC channels contribute to the myogenic response of afferent arterioles (Wang et al. 2008; Guan et al. 2009). Renal interlobar, mesenteric, and middle cerebral arteries myogenic responses were also dependent on ENaC proteins (Jernigan and Drummond 2005; Jernigan et al. 2008; Kim et al. 2012). The goal of this study was to determine if ENaC channels contributed to the afferent arteriolar vasoconstriction to P2X receptor activation or 20-HETE.

There is significant evidence in renal epithelial cells that ATP and P2X receptor activation can alter ENaC activity and that feedback interactions between P2X receptors and ENaC exist (Wildman et al. 2005, 2009; Zhang et al. 2007). In renal collecting duct cells ATP through P2X receptor activation inhibits apical ENaC mediated sodium influx and ENaC increases the plasma membrane expression of P2X receptors (Wildman et al. 2005). Conversely, ATP and P2X receptor agonists including β,γ-methylene ATP stimulate the opening of amiloride-sensitive ENaC and increases sodium transport in A6 cells (Zhang et al. 2007). P2X receptors also share an intriguing structural relatedness to DEG/ENaC channels and have been demonstrated to be involved in mechanotransduction processes (Di Virgilio et al. 1998; Shinoda et al. 2009; Browne et al. 2010; Kessler et al. 2011). Based on results in epithelial cells, it has been suggested that P2X receptors and ENaC might be in close physical proximity on the plasma membrane (Zhang et al. 2007; Wildman et al. 2009). Taken together, these findings provide evidence that P2X receptor activation could result in increased afferent arteriolar DEG/ENaC activity resulting in vasoconstriction. Findings of this study demonstrate that the afferent arteriolar vasoconstriction in response to P2X receptor activation was unaltered by the ENaC inhibitors amiloride or benzamil.

Although there is significant data linking P2X receptors to ENaC activity, there is less known data about 20-HETE interactions with DEG/ENaC channels. ENaC activity was slightly inhibited by 20-HETE in cultured mouse cortical collecting duct cells (Wang et al. 2009). Experimental studies using hydroxylase inhibitors to decrease 20-HETE levels or expression of CYP4A10 to increase 20-HETE levels were unable to detect changes in ENaC activity in cortical collecting duct cells (Wei et al. 2004; Pavlov et al. 2011). Other arachidonic acid metabolites have been demonstrated to decrease or increase ENaC activity. Epoxyeicosatrienoic acids (EETs) have been consistently demonstrated to decrease renal epithelial cell ENaC activity whereas prostaglandins (PG) such as PGE2 and PGF2α have been demonstrated to increase ENaC activity (Wei et al. 2004; Wang et al. 2009; Pavlov et al. 2011). Thus, there is the possibility that 20-HETE could cause vasoconstriction of afferent arterioles through DEG/ENaC activation. In this study, 20-HETE afferent arteriolar vasoconstriction was not altered by ENaC inhibition.

Overall, the results of this study revealed that ENaC activation in afferent arterioles is not downstream of P2X receptor activation or 20-HETE. However, it is still possible that DEG/ENaC channels activate P2X receptors or increase 20-HETE levels. This possibility could not be tested as DEG/ENaC channel agonists are not available. Experimental studies utilizing DEG/ENaC inhibitors and measuring afferent arteriolar ATP or 20-HETE release could potentially test this possibility. An alternative explanation is that P2X receptor activation and 20-HETE contribute to tubuloglomerular feedback responses whereas DEG/ENaC channels participate in myogenic responses. Further studies and novel experimental tools will be needed to address this issue.

In summary, ENaC inhibitors amiloride and benzamil attenuated the afferent arteriolar response to increasing perfusion pressure but did not alter the vasoconstrictor responses to β,γ-methylene ATP or 20-HETE. Therefore, we conclude that DEG/ENaC channels are required for afferent arteriolar autoregulatory responses but do not contribute to P2X receptor- or 20-HETE-mediated afferent arteriolar vasoconstriction.
